# Partial resection of large congenital left ventricular diverticulum in an infant: a case report

**DOI:** 10.1186/s12893-020-00808-6

**Published:** 2020-06-30

**Authors:** Yibing Fang, Qi An, Tianping Yu, Shuhua Luo

**Affiliations:** 1 Department of Cardiovascular Surgery, Sichuan University West China Hospital, #37 Guoxue Xiang, Chengdu, 610041 Sichuan China; 2 Department of Pathology, Sichuan University West China Hospital, Chengdu, China

**Keywords:** Partial resection, Congenital left ventricular diverticulum, Infant, Case report

## Abstract

**Background:**

Congenital left ventricular diverticulum is a rare cardiac malformation usually requiring total resection.

**Case presentation:**

This report describes an infant presenting with a large apical diverticulum with a wide ventricle connection. Given the vicinity of the left anterior descending coronary artery to the diverticulum and its wide ventricular connection, partial resection was undertaken. The patient remained asymptomatic with good heart function 8 months after surgery. The last follow-up echocardiography did not demonstrate any significant left ventricular outpouching.

**Conclusions:**

We advocate early treatment of left ventricular diverticulum in children given the risk of spontaneous rupture of diverticulum, sudden death, and other serious complications if left untreated. For small patients with a wide connection of diverticulum to ventricle, partial resection is a safe option with favorable short-term outcomes.

## Background

Congenital left ventricular diverticulum (LVD) is a rare congenital malformation with a prevalence of 0.4% according to one series of 750 autopsies of congenital cardiac defect [1]. LVD is commonly diagnosed during early childhood because of its frequent association with other cardiac and midline thoraco-abdominal anomalies [2]. In a majority of cases, the ratio of the diameter of the connection with the left ventricular cavity (the “neck” of the diverticulum) to the maximum diameter of the diverticulum body is usually smaller than 1 [3]. Here we report the case of an infant with apical LVD and an unusually wide “neck”, who underwent successful surgical partial resection. Clinical presentation, surgical methods, and short-term outcomes are discussed.

## Case presentation

**Fig. 1:**
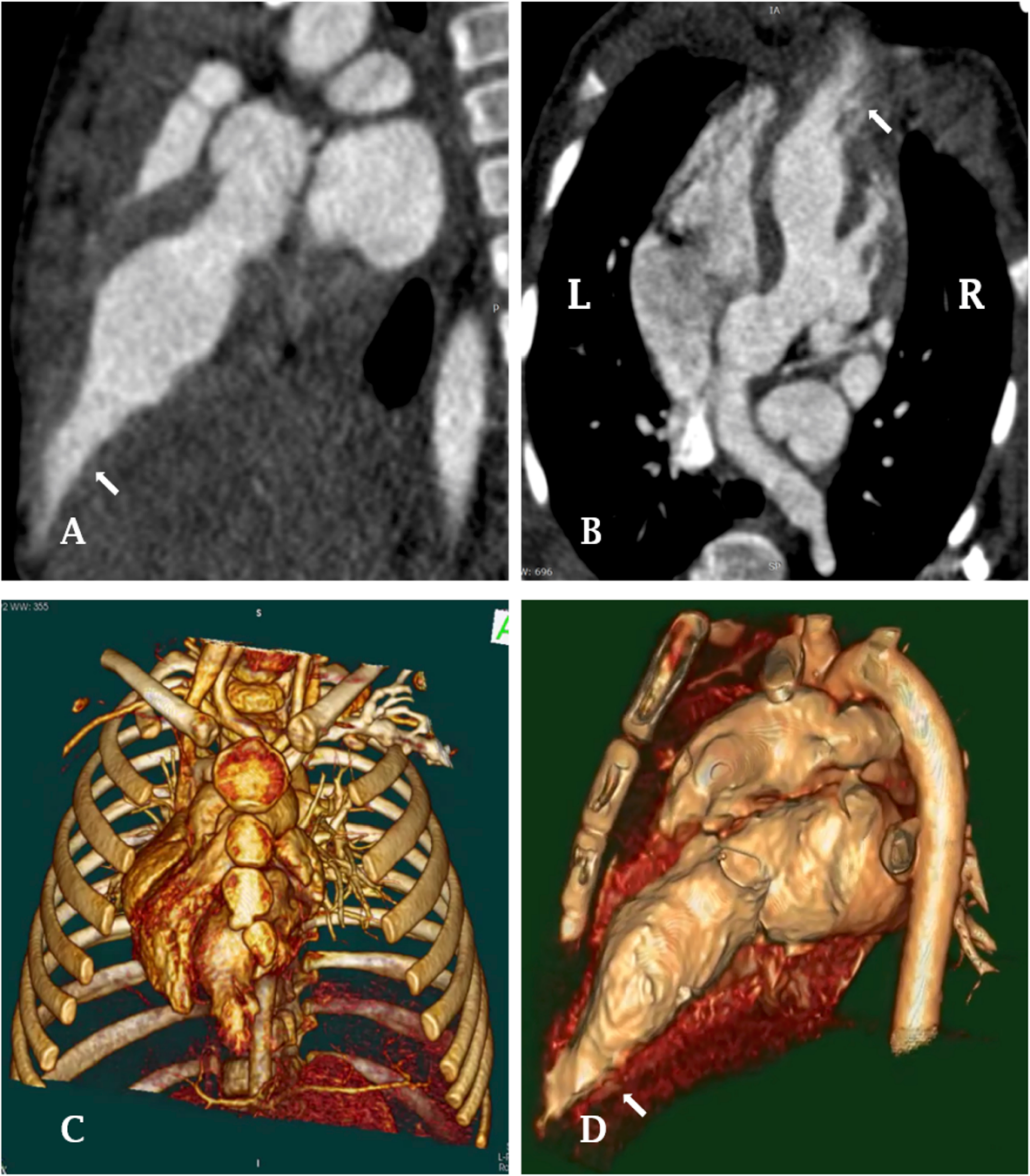
Preoperative computed tomography images of the diverticulum. Images showing a protrusion of the left ventricle (arrow) extending beyond the apex, with a wide ventricle connection (**a**: sagittal view; **b**: coronal view). A three-dimensional reconstruction showing the morphology (**c**) and position (**d**) of the diverticulum

A 9-month-old female infant (11.7 kg) was admitted to our hospital for a previously undiagnosed growing pulsatile mass at the lower chest that was present since birth and, according to family, had been gradually growing (Video 1). She was otherwise asymptomatic, with no history of embolic events, syncope, arrhythmia, or heart failure. Physical examination revealed aplasia of the xiphoid process and lower sternum (Video 1). The preoperative electrocardiogram was normal. Transthoracic echocardiography showed dextrocardia with the apex pointing to the right side, and a large contractile pouch arising from the apex, contracting in synchrony with the left ventricle (Video 2). No other heart defects were identified. Enhanced computed tomography (CT) confirmed a protrusion extending beyond the apex and showing a wide connection with the left ventricular cavity (Fig. 1). The infant was diagnosed with LVD and treated immediately by surgery, without a period of prior observation or conservative management, because it was growing.

**Fig. 2:**
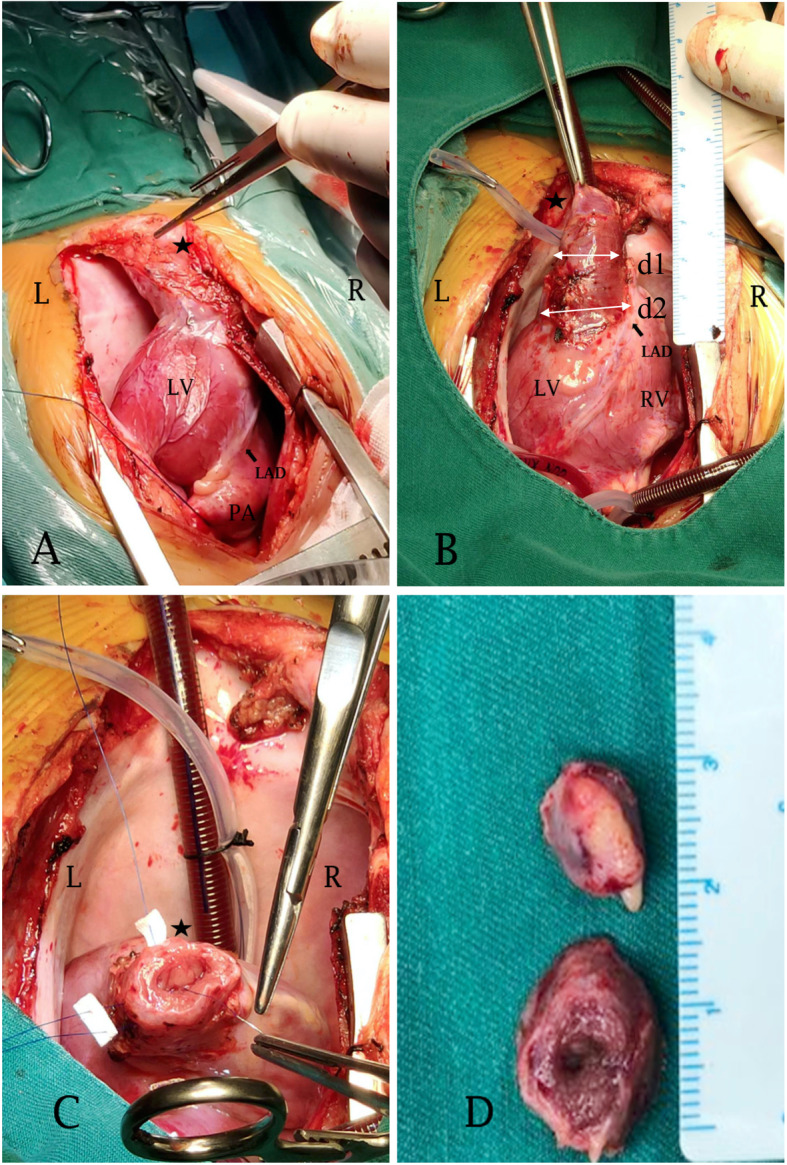
Intraoperative exploration (**a**-**c**) and partial resection (**d**) of the diverticulum. Panel **b** shows, under direct visualization, the ratio between the diverticulum connection with the left ventricular cavity (d2 = 4 cm) and the maximum diameter of the body of the diverticulum (d1 = 3 cm) was 1.3. Abbreviations: LV: left ventricle; RV: right ventricle; LAD: left anterior descending coronary artery; PA: pulmonary artery

The patient underwent surgical resection using cardiopulmonary bypass. Under direct visualization, the ratio of the diameter of the diverticulum “neck” (4 cm) to the maximum diameter of the diverticulum body (3 cm) was 1.3 (Fig. 2a and b). In addition, the left anterior descending coronary artery (LAD) was close to the ventricular connection (Fig. 2a and b). Given the vicinity of the LAD to the LVD and the wide ventricular connection, partial resection was undertaken by excising the distal half of the diverticulum, and the remnant was oversewn using an interrupted pledgeted 5–0 polypropylene suture (Prolene, Ethicon, USA) (Fig. 2c). The right pericardium was resected carefully to avoid damaging the phrenic nerve, and the left ventricular apex was successfully repositioned to the right thoracic cavity.

**Fig. 3:**
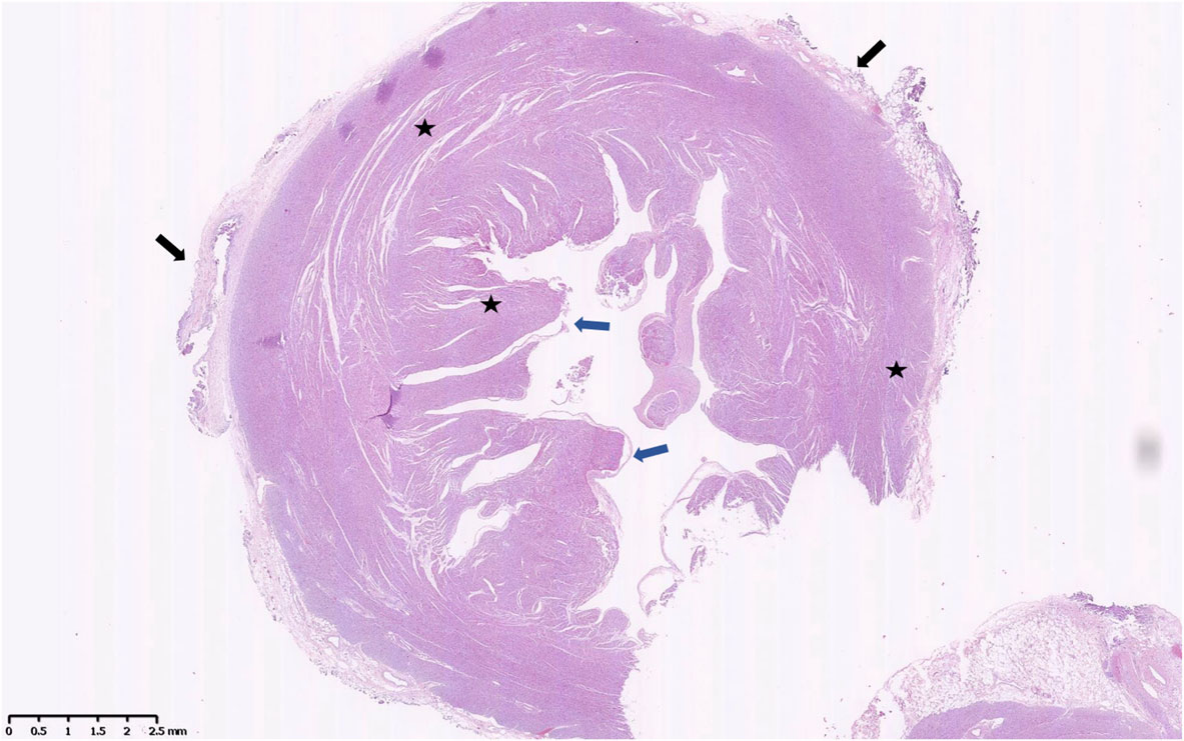
Histopathology of the resected diverticulum. Shown are the epicardium (external layer, black arrow), myocardium (middle layer, star) and endocardium (inner layer, blue arrow)

The postoperative course was unremarkable. Histopathology of surgical specimens showed the presence of all three layers of the heart (endocardium, myocardium, and epicardium), and no fibrotic tissue was identified anywhere in the lesion (Fig. 3). During 8-month follow-up, the infant remained asymptomatic with good heart function. The last follow-up echocardiography showed normal left ventricular geometry without obvious evidence of apical outpouching. No thrombosis was identified (Video 3).

**Additional file 3: Video 3.** Follow-up transthoracic echocardiogram (four-chamber view). The normal left ventricle geometry without obvious evidence of apical outpouching is observed.

## Discussion and conclusions

A typical LVD involves a narrow connection with the left ventricle and a normally developed and contracting wall [3]. Differential diagnosis of LVD should take into account two other types of congenital left ventricular outpouching: left ventricular aneurysm and double-chambered left ventricle. In contrast to LVD, left ventricular aneurysm involves a broad connection neck and the absence of some of the layers in the aneurysm wall [4]. A double-chambered left ventricle involves the presence of two ventricular cavities (main chamber and accessory chamber), separated by an abnormal muscle band. The accessory chamber shares a wide connection to the main chamber as in an aneurysm, but it contains all three myocardium layers as in a diverticulum. The accessory chamber usually has a thinned, poorly contracting wall with decreased systolic function [5, 6]. In our patient, the abnormal left ventricular outpouching showed the wide connection common in left ventricular aneurysm and double-chambered left ventricle, but the left ventricle showed no abnormal muscle band. Moreover, the wall of the outpouching showed normal contractility and thickness, and it contained the three layers of the myocardium without signs of fibrosis. These considerations, together with the associated midline thoraco-abdominal defect, led us to diagnose LVD.

In our opinion, the decision about surgical resection requires a thorough, individualized assessment of the patient, including age, thickness of the diverticulum wall, location of diverticulum, presence of arrythmia and systemic embolism, changes in size over time, heart function and associated intracardiac anomalies. A novel grading system was recently proposed for defining the size of all left ventricular outpouching including diverticulum, based on circumference, area, and volume indices [4]. There is insufficient evidence to support the relationship between diverticulum size and risk of complications, such as spontaneous rupture of diverticulum; however, it may be reasonable to conservatively manage adult patients with small, asymptomatic LVD.

In a comprehensive study analyzing the clinical outcomes in 453 published patients with LVD, 2.9% had embolic events, 9.9% presented arrhythmia, and 6.8% showed complications with heart failure [3]. During follow-up, the leading cause of cardiac death in LVD patients was spontaneous rupture of diverticulum (6/8, 75%), which occurred only in patients younger than 8 years old [3]. A case series of 12 neonatal patients with LVD or left ventricular aneurysm undergoing conservative management reported that 10 patients experienced at least one adverse event, including 2 spontaneous rupture of diverticulum [7]. Although consensus is lacking on the optimal management of asymptomatic LVD, in our institution we tend to surgically resect the diverticulum in children, since rupture and other serious complications appears to be a problem among younger patients [3].

Perioperative mortality may occur in approximately 7.0% of LVD patients [3]. Outcomes of pediatric LVD are generally good after repair, although prognosis depends on associated intracardiac malformations [7]. One study reported four pediatric patients who remained free of symptoms during a mean follow-up of 10 years [8]. The most appropriate technique for surgical resection depends on LVD location and size. We agree with others [3] that resection by direct suturing may be the best option when the connection to the left ventricle is shorter than 2 cm. For those with a wide connection to the left ventricle, the best surgical option may be resection of the entire diverticulum and insertion of various types of patch closures.

We performed partial resection in our patient since we were concerned about left ventricular dysfunction if we resected the entire diverticular wall containing normally contracting myocardium in a relatively small patient. The vicinity of the LAD to the diverticulum also made us concerned about the risk of coronary artery kinking or narrowing in the case of total resection.

To our knowledge, transcatheter device closure of LVD has been reported in only two patients, both of whom had favorable outcomes [9, 10] We suspect the transcatheter intervention may require an even smaller connection than direct suturing, such as 0.4 and 0.13 cm in studies of two patients with good outcomes after the transcatheter procedure [9, 10]. In the present case, preoperative multidisciplinary discussion including two senior interventional cardiologists resulted in consensus for surgical resection, as the wide diverticulum connection was judged unsuitable for transcatheter intervention.

In our patient, the future risk of arrhythmia is low due to the lack of fibrotic tissue in the diverticulum. Yearly echocardiography and 24-h Holter monitoring are planned for long-term follow-up, and magnetic resonance imaging will be used if necessary to assess regression or enlargement of the remnant left ventricle.

In conclusion, we present an infant patient with asymptomatic congenital apical LVD associated with an unusually wide connection. Given the unusual morphology of the diverticulum and the patient’s small body, partial resection was undertaken with favorable short-term outcomes.

## Supplementary information

**Additional file 1: Video 1.** Inspection of the left ventricular diverticulum.

**Additional file 2: Video 2.** Preoperative transthoracic echocardiogram (four-chamber view). A large contractile pouch arising from the apex, contracting in synchrony with the left ventricle, is observed.

## Data Availability

The data supporting the findings of this study are available within the article.

## Abbreviations

LVD: Left ventricular diverticulum
LAD: Left anterior descending coronary artery
